# Mesenchymal stem cells improve redox homeostasis and mitochondrial respiration in fibroblast cell lines with pathogenic *MT-ND3* and *MT-ND6* variants

**DOI:** 10.1186/s13287-022-02932-x

**Published:** 2022-06-17

**Authors:** Tharsini Navaratnarajah, Marlen Bellmann, Annette Seibt, Ruchika Anand, Özer Degistirici, Roland Meisel, Ertan Mayatepek, Andreas Reichert, Fabian Baertling, Felix Distelmaier

**Affiliations:** 1 Department of General Pediatrics, Neonatology and Pediatric Cardiology, University Children’s Hospital, Medical Faculty, Heinrich Heine University Düsseldorf, Moorenstr. 5, 40225 Düsseldorf, Germany; 2 Institute of Biochemistry and Molecular Biology I, Medical Faculty and University Hospital Düsseldorf, Heinrich Heine University Düsseldorf, Düsseldorf, Germany; 3 Division of Pediatric Stem Cell Therapy, Department of Pediatric Oncology, Hematology and Clinical Immunology, Medical Faculty, Heinrich Heine University Düsseldorf, Düsseldorf, Germany

**Keywords:** Mitochondrial DNA, Mesenchymal stem cells, Complex I, Gene therapy, Mitochondrial transfer, ND3, ND6

## Abstract

The most frequent biochemical defect of inherited mitochondrial disease is isolated complex I deficiency. There is no cure for this disorder, and treatment is mainly supportive. In this study, we investigated the effects of human mesenchymal stem cells (MSCs) on skin fibroblast derived from three individuals with complex I deficiency carrying different pathogenic variants in mitochondrial DNA-encoded subunits (*MT-ND3, MT-ND6*). Complex I-deficient fibroblasts were transiently co-cultured with bone marrow-derived MSCs. Mitochondrial transfer was analysed by fluorescence labelling and validated by Sanger sequencing. Levels of reactive oxygen species (ROS) were measured using MitoSOX Red. Moreover, mitochondrial respiration was analysed by Seahorse XFe96 Extracellular Flux Analyzer. Levels of antioxidant proteins were investigated via immunoblotting. Co-culturing of complex I-deficient fibroblast with MSCs lowered cellular ROS levels. The effect on ROS production was more sustained compared to treatment of patient fibroblasts with culture medium derived from MSC cultures. Investigation of cellular antioxidant defence systems revealed an upregulation of SOD2 (superoxide dismutase 2, mitochondrial) and HO-1 (heme oxygenase 1) in patient-derived cell lines. This adaptive response was normalised upon MSC treatment. Moreover, Seahorse experiments revealed a significant improvement of mitochondrial respiration, indicating a mitigation of the oxidative phosphorylation defect. Experiments with repetitive MSC co-culture at two consecutive time points enhanced this effect. Our study indicates that MSC-based treatment approaches might constitute an interesting option for patients with mitochondrial DNA-encoded mitochondrial diseases. We suggest that this strategy may prove more promising for defects caused by mitochondrial DNA variants compared to nuclear-encoded defects.

**Supplementary Information:**

The online version contains supplementary material available at 10.1186/s13287-022-02932-x.

## Background

Mitochondrial oxidative phosphorylation (OXPHOS) delivers the overall energy that is required to enable cell functioning. During the last decades, numerous genetic defects affecting components of the OXPHOS system emerged as a cause of severe neurodegenerative diseases [1–4]. The underlying pathophysiology includes impaired energy production and oxidative stress [5]. The most common cause of mitochondrial disease is isolated complex I (CI) deficiency [6, 7]. CI is the largest OXPHOS complex and consists of 44 different protein subunits, which are encoded by the mitochondrial and the nuclear genome [8]. Currently, there is no cure available for these disorders.

Mesenchymal stem cells (MSCs) are subject to a wide range of research related to cell-based treatment for human diseases [9, 10]. Recent studies revealed the ability of intercellular mitochondrial transfer from MSCs to different types of cells through formation of tunnelling nanotubes or cell junctions [11–18].

We have previously demonstrated that co-culturing allows human MSCs to transfer mitochondria to fibroblasts with nuclear-encoded CI deficiency due to *NDUFS4* mutation [11]. We observed beneficial effects on redox homeostasis. However, this improvement was only mild and transient. We hypothesised that this limited effect is related to the fact that NDUFS4 is a nuclear-encoded CI subunit. Accordingly, MSC-based mitochondrial transfer will only provide a short-lasting effect on the protein level. In contrast, MSC treatment of cell lines with pathogenic mitochondrial DNA (mtDNA)-variants might constitute a more promising model system since wild-type mtDNA will be transferred to the defective cell lines [19, 20].

## Materials and methods

### Cell lines

Patient-derived fibroblast lines carried variants in the *MT-ND3* gene (MT-ND3a and MT-ND3b; m.10191T>C and m.10197G>A, respectively) or in the *MT-ND6* gene (MT-ND6; m.14487T>C). Primary human skin fibroblasts (NHDF-neo, Lonza) were used as controls. Cell lines were cultured, and human MSCs were isolated, cultured and selected as previously described [11]. The use of patient-derived cell lines was approved by the local ethics committee (#4161).

### Fluorescence labelling

Fluorescence labelling was performed as previously described [11]. Briefly, mitochondrial cytochrome *c* oxidase subunit 8A (Cox8a)-GFP and lamin B1 (LMNB)-RFP/BFP were amplified from pENTR vectors (OriGene) via PCR and subcloned individually into pLenti6.3/V5-TOPO vector using the pLenti6.3/V5-TOPO TA cloning Kit (Invitrogen), which contains a blasticidin resistance marker for selection.

### Co-culturing

Co-culturing was performed as described previously [11]. For seahorse, ROS and sequence analysis, transduced fibroblasts with blasticidin resistance were co-cultured with non-transduced MSCs and, for mitochondrial transfer analysis, with transduced MSCs in a 1:1 ratio for 72 h in MSC medium (without blasticidin). MSC elimination was performed with selection medium for 10 days (3 µg/ml blasticidin; Gibco by life technologies). For sequential MSC treatment, 72 h co-culturing and 10 day MSC elimination was repeated. In another approach to analyse the supernatant effect, co-culture was performed in a transwell (Corning) system with MSCs seeded at 1.5 × 10^5^ on plate inserts with polycarbonate membrane of 0.4 µm pore size and fibroblasts seeded at 2 × 10^5^ in the well below.

### Analysis of mitochondrial transfer

Co-cultured cells were detached and fixed 20 min with fixation buffer (BioLegend). Subsequently, cells were analysed by LSR Fortessa flow cytometer (BD Biosciences). Gating was performed as previously described [11]. Data analysis was performed using FlowJo software (FlowJo v10, LLC).

### mtDNA analysis

For mtDNA sequencing, first, total DNA (QIAamp DNA Mini Kit; Qiagen) and, then, mtDNA (REPLI-g mitochondrial DNA Kit; Qiagen) were isolated from fibroblasts, MSCs and co-cultured cells according to the manufacturer’s protocols. Amplification of the mitochondrial hypervariable region I and II was performed using Phusion High-Fidelity DNA polymerase (Thermo scientific) and following primers: forward 5′-TCTCCGATCCGTCCCTAACA-3′; reverse 5′-GGCACGAAATTGACCAACCC-3′. PCR products were purified using Nucleospin gel and PCR clean-up kit (Macherey–Nagel). Sequences were analysed by Sanger sequencing (Eurofins).

## MSC supernatant preparation

Preparation of supernatant was performed as described previously [16]. At least 3-day-old MSC culture medium was collected and separated from cell debris (2000×*g*, 10 min). Supernatant was added to fibroblasts and replaced every 24 h over a period of 72 h.

### Measurements of reactive oxygen species

Mitochondrial reactive oxygen species (ROS) production was determined as previously described [21]. Briefly, cells were treated with medium containing MitoSOX Red (5 nM, 10 min, 37 °C; Invitrogen) and analysed with Axio Observer Z1 microscope (Zeiss). Image analysis was performed using ImageJ software (http://rsbweb.nih.gov/ij/).

### SDS PAGE and Western Blotting

After co-culture, cell pellets were collected and frozen at − 80 °C. Then, pellets were thawed, and nuclear and cytosolic fractions were extracted according to manufacturer’s description of the NE-PER Nuclear and Cytoplasmic Extraction Reagents Kit (Thermo Scientific Fisher). Protein levels were quantified using the BCA Protein Assay Reagent Kit (Thermo Scientific Fisher). Protein lysates (20 µg) were separated on 4–12% Bis–Tris gels (Thermo Scientific Fisher), transferred to PVDF membranes and decorated with antibodies as indicated. Signals were visualised using ECL solutions (Roche) and ChemiDoc Touch Imaging System (Bio-Rad). Blots were stripped and reblotted. Intensity of bands was quantified by Image Lab v6.1.0 software (Bio-Rad) and normalised to untreated control. However, for quantification of SOD2 levels, data were normalised to the levels of MT-ND3a as there was nearly no SOD2 detectable for controls. SOD2 data were then normalised to SDHA, HO-1, NQO1 and cytosolic fractions were normalised to GAPDH and nuclear fractions were normalised to HDAC1. Data were obtained from at least three independent experiments. Data are expressed as mean ± SD. Statistical analysis was performed with unpaired Student’s *t* test, *p < 0.05, **p < 0.01, ***p < 0.001. Following antibodies were used: α-Nrf2 (Proteintech #16396-1-AP), α-HDAC1 (Cell Signaling #34589), α-GAPDH (Thermo Scientific Fisher #AM4300), α-SDHA (Abcam #ab14715), α-α-Tubulin (Sigma Aldrich, T-9026), α-SOD2 (Proteintech #66474-1-Ig), α-NQO1 (Proteintech #11451-1-AP), α-HO-1 (Proteintech #66743-1-Ig).

### Mitochondrial respiration

Mitochondrial respiration was analysed by measuring the oxygen consumption rate (OCR) with a Seahorse XFe96 Extracellular Flux Analyzer and XF Cell Mito Stress Test Kit (Agilent) according to manufacturer’s instructions with few changes.

Briefly, 24 h prior to measurement, 18.000 cells/well were seeded in 96-well Seahorse XF and grown at 37 °C and 5% humidified CO_2_. Sensor cartridge was hydrated in XF Calibrant overnight at 37 °C and no CO_2_. On day of measurement, cells were washed once and growth medium was replaced with unbuffered XF DMEM Medium, pH 7.4 (supplemented with 25 mM D-glucose, 4 mM L-glutamine and 1 mM sodium pyruvate), followed by approximately 50 min incubation at 37 °C without CO_2_. OCR was measured under basal conditions and after sequential injection of 1.5 µM oligomycin, 2 µM FCCP and 0.5 µM rotenone/antimycin A (Rot/AA). Subsequently, cell numbers/well were determined by Hoechst staining. Data were analysed using wave software (Agilent) and are expressed as the OCR in pmol/min/cell or the extracellular acidification rate (ECAR) in mpH/min, normalised to cell number in each well. All data were further normalised to basal respiration of untreated control.

### Statistical analysis

For statistical analysis, *t* test was performed using GraphPad Prism version 7.05 (San Diego, CA, USA).

## Results

### MSCs transfer mitochondria to CI-deficient fibroblasts

**Fig. 1:**
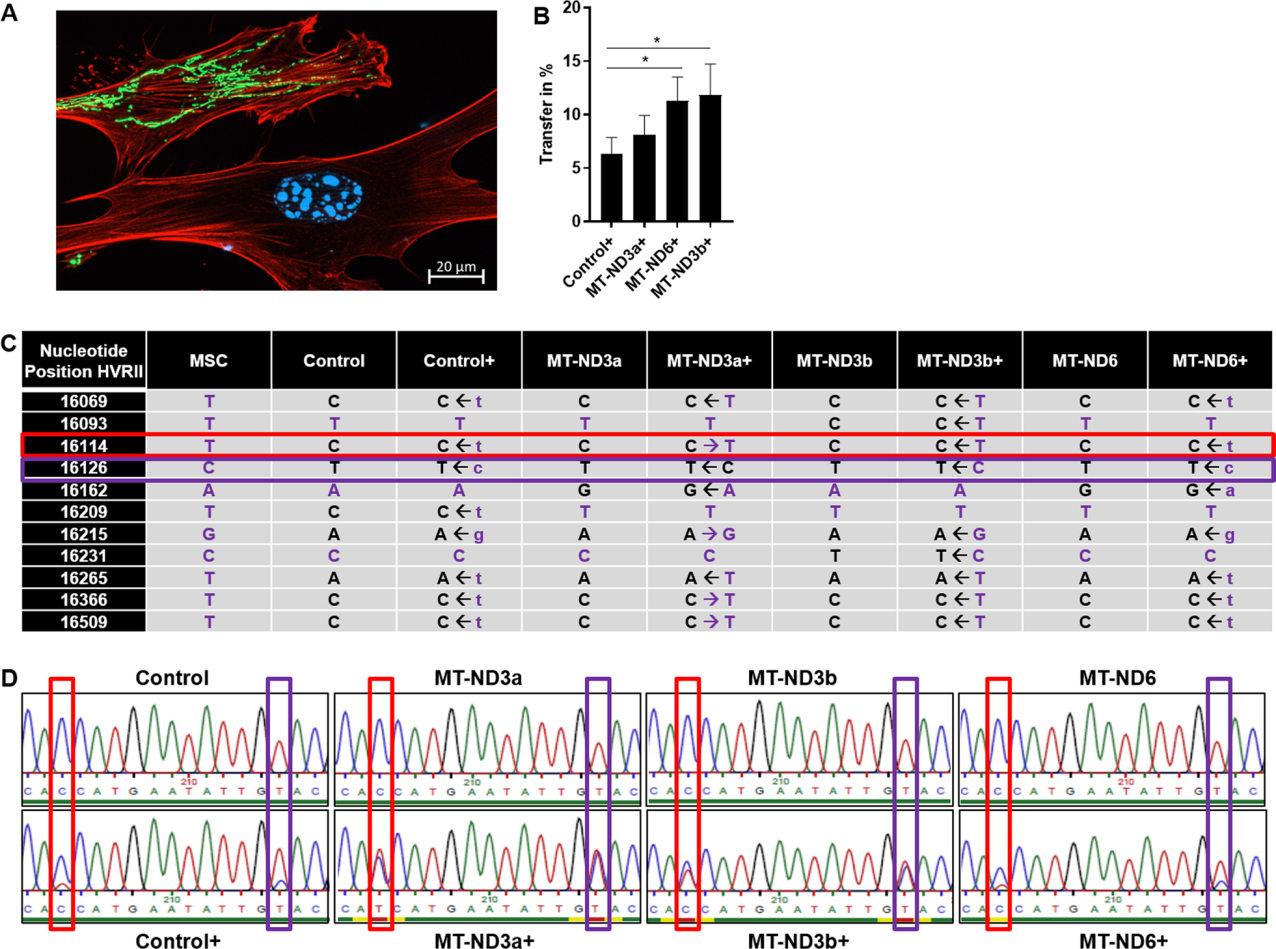
Transfer of mitochondria and mitochondrial DNA from MSCs to fibroblasts. **A** Representative image of MSCs with GFP-tagged mitochondrial Cox8a and fibroblasts with BFP-tagged nuclear-located LMNB. To visualise cell structure, cells were additionally stained with Alexa Fluor 594 phalloidin (Thermo Scientific). **B** Quantitative flow cytometry analysis of mitochondrial transfer between fibroblasts and mesenchymal stem cells (MSCs). Mitochondrial transfer rate was measured and quantified after 72 h of co-culturing. MSCs were either co-cultured with healthy control fibroblasts (Control+) or patient fibroblasts carrying either the *MT-ND6* variant (MT-ND6+), the m.10191T>C variant in the *MT-ND3* gene (MT-ND3a+) or the m.10197G>A variant in the *MT-ND3* gene (MT-ND3b+). The percentage (%) of fibroblasts positive for Cox8a-GFP and LMNB-RFP in relation to all LMNB-RFP positive cells are shown. Data are displayed as mean and error bars indicate standard error of the mean (SEM). *p < 0.05. **C** mtDNA sequencing of hypervariable regions in control and patient fibroblasts after co-culturing with MSCs. Bases at specific nucleotide positions indicated as position in the entire mitochondrial genome are shown in mesenchymal stem cells (MSC) as well as in control fibroblasts (Control) and fibroblasts carrying variants in either *MT-ND6* (MT-ND6) or in *MT-ND3* (m.10191T>C—MT-ND3a; m.10197G>A—MT-ND3b). Control and patient fibroblasts after 72 h hours of co-culturing with MSCs and subsequent removal of MSCs are indicated by “+ ” (e.g. “MT-ND3a+”). MSCs-treated fibroblasts mainly show changes of mtDNA in hypervariable region II (HVRII). Treated fibroblasts contain double peaks in the chromatograms (**D**) that vary in height. Arrows (**C**) indicate which peak is bigger, and further capital letters are used to indicate comparable peak heights, while small letters are used to indicate smaller peaks. Purple letters represent MSC mtDNA sequence. **D** Representative segments of chromatograms of sequenced regions of untreated and MSC-treated (+) control and patient fibroblasts after Sanger sequencing. Treated fibroblasts show second peaks that derive from MSC mtDNA. Nucleotide positions with two peaks are highlighted by red (position 16114 of the mtDNA) or purple box (position 16126 of the mtDNA)

CI-deficient and control fibroblasts with LMNB-RFP- or LMNB-BFP-labelled nuclei and MSCs with Cox8a-GFP-labelled mitochondria were co-cultured for 72 h (for example, see Fig. 1A). After co-cultivation, a subpopulation of LMNB-RFP/BFP and Cox8a-GFP double positive cells was observed in control as well as CI-deficient fibroblasts, which demonstrates mitochondrial transfer from MSCs to fibroblasts. In all CI-deficient lines, transfer of mitochondria from MSCs ranged between 8 and 11.9%. Transfer rate from MSCs to control fibroblasts was 6.3% (Fig. 1B).

## MSC mtDNA is detectable in CI-deficient fibroblasts

mtDNA of treated and untreated fibroblasts as well as MSCs was isolated, and the hypervariable regions I and II (HVR I + II) were amplified via PCR. Bases within these regions typically differ between cell lines. After Sanger sequencing, base differences between MSCs and untreated fibroblasts were detected within both hypervariable regions (Fig. 1C, D). Sequence analysis of MSC-treated fibroblasts revealed that they contained both bases at the respective positions (Fig. 1C, D), which proves that mtDNA of MSCs is still present 10 days after co-cultivation with MSCs.

## MSC co-culture leads to a sustained reduction of ROS production in CI-deficient fibroblasts

**Fig. 2:**
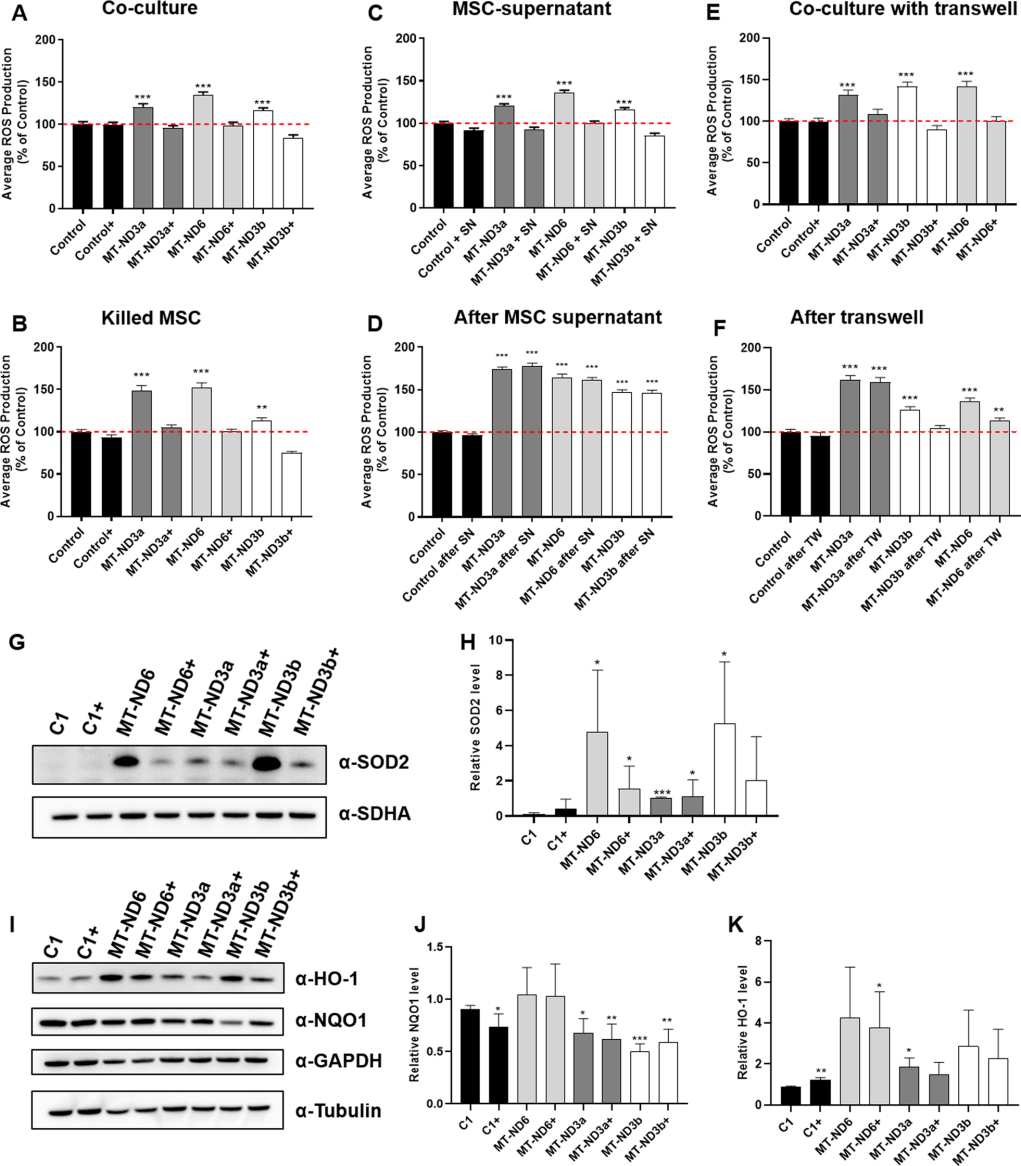
Mitochondrial ROS production in control and patient fibroblasts after co-culturing with mesenchymal stem cells (MSC) or treatment with MSC supernatant. **A** ROS production as percentage (%) fluorescence intensity of control under steady-state conditions in control fibroblasts (Control) or patient fibroblasts carrying variants in either *MT-ND6* (MT-ND6) or in *MT-ND3* (m.10191T>C—MT-ND3a; m.10197G>A MT-ND3b) and after 72 h of co-cultivation with MSCs (+). ROS level elevation in patient cells is mitigated by co-culturing with MSCs. **B** ROS production under steady-state conditions and 10 days after removal of MSCs from the co-culturing system by blasticidin treatment. The mitigation of ROS level elevation persists even after MSC removal. **C** ROS production under steady-state conditions and after 72 h treatment with MSC supernatant (+SN). Supernatant treatment reduces ROS production in patient fibroblasts. **D** ROS production under steady-state conditions and after 72 h treatment with MSC supernatant followed by subsequent removal of MSC supernatant (SN) and culturing with regular medium (after SN) for 8 days. After removal of MSC supernatant, ROS production is similar to that under steady-state conditions. **E** ROS production under steady-state conditions compared to 72 h treatment with MSCs in a transwell (+TW) system. Paracrine effects of MSCs lead to reduction of ROS levels in patient fibroblasts. **F** ROS production under steady-state conditions and after 72 h treatment with MSCs in transwell system followed by subsequent removal of insert plate and culturing with regular medium (after TW) for 8 days. After removal of MSC, ROS production reduced to a lesser extent in patient MT-ND3b and MT-ND6. MT-ND3a levels are similar to untreated levels. Date are shown as mean ± SEM. **p < 0.01, ***p < 0.001. **G** Representative Western Blot results from cytosolic fractions analysed for SOD2 and SDHA protein levels in cells with (+) and without MSC treatment. **H** Quantification of SOD2 levels using the original blots from five independent experiments. Data are expressed as mean ± SD. *p < 0.05, ***p < 0.001. **I** Representative Western Blot results from cytosolic fractions analysed for HO-1, NQO1, GAPDH and α-Tubulin protein levels in cells with (+) and without MSC treatment. **J** Quantification of NQO1 levels using the original blots from four independent experiments. Data are expressed as mean ± SD. *p < 0.05, **p < 0.01, ***p < 0.001. **K** Quantification of HO-1 levels using the original blots from three independent experiments. Data are expressed as mean ± SD. *p < 0.05, **p < 0.01

ROS are produced as a by-product of mitochondrial electron transfer and are typically increased in CI deficiency [22–24]. ROS production was quantified using fluorescent labelling with MitoSOX Red. All CI-deficient cell lines displayed increased ROS production (Fig. 2A). Next, ROS production was measured after co-cultivation of CI-deficient fibroblasts with MSCs. To distinguish cell types, mitochondria in MSCs were labelled with Cox8a-GFP and the nuclei of fibroblasts were labelled with LMNB-BFP (Fig. 1A). In these experiments, ROS production of CI-deficient cell lines was reduced to control levels (Fig. 2A). This effect on ROS production persisted after selective removal of MSCs (Fig. 2B). In further experiments, patient fibroblasts were treated with MSC medium supernatant for 72 h with subsequent measurement of ROS production. This treatment also led to a reduction of ROS production to control levels (Fig. 2C). However, after further culturing for 8 days in regular medium ROS production increased to pre-treatment levels (Fig. 2D). The same experiment was repeated in a transwell culture system to analyse the effects of MSCs in a setting in which cells are in permanent co-culture but without the possibility of direct cell–cell interaction. Again, subsequent measurement following co-culture led to reduction of ROS to control levels (Fig. 2E). Interestingly, 8 days after termination of transwell culture and cell culture in regular medium the effect persisted in patient fibroblasts MT-ND3b and MT-ND6, however, to a lesser extent and not in the MT-ND3a cell line (Fig. 2F).

In another approach, free glutathione to oxidised glutathione ratio was measured. Only patient MT-ND3a showed increased total glutathione levels, which decreased to control levels after MSC treatment (Additional file 2: Fig. S1A). However, the GSH/GSSG ratio was similar in all analysed cells and unchanged between treated and untreated fibroblasts (Additional file 2: Fig. S1B).

## MSC co-culture alters the levels of antioxidant proteins

Increased ROS can be neutralised by various cellular antioxidant defence systems. Most antioxidant genes are regulated by nuclear factor erythroid 2-related factor 2 (Nrf2), a master-regulator of the oxidative stress response. In our model system, we could not observe a MSC treatment-affected regulation of Nrf2 in cytosol or nucleus (Additional file 2: Fig. S1C–E). We further analysed Nrf2-target genes, such as SOD2, NQO1 and HO-1. While cellular GSH is needed to reverse lipid peroxidation reactions and removal of hydrogen peroxide (H_2_O_2_), superoxide dismutases (SODs) covert oxygen radicals into H_2_O_2_. Mitochondrial SOD, also known as SOD2, is found in the mitochondrial matrix and defends the cell against accumulating ROS. Visual and quantitative analysis of SOD2 levels reveals that levels were elevated compared to healthy controls in untreated patient fibroblasts, especially in MT-ND6 and MT-ND3b fibroblasts (Fig. 2G–H). After MSC treatment, SOD2 levels were clearly reduced in patient fibroblasts of MT-ND6 and MT-ND3b, but also in fibroblast line MT-ND3a. However, reduction was not statistically significant due to high variance between experiments. NAD(P)H dehydrogenase (quinone) 1 (NQO1) is responsible for the reduction of NADPH oxidase activity and ROS secretion. NQO1 levels were not altered by MSC treatment (Fig. 2I, J). Nrf2-dependent heme oxygenase 1 (HO-1) was increased in untreated patient fibroblasts compared to healthy controls; however, differences were not statistically significant (Fig. 2I, K). After MSC treatment, levels appeared to be decreased in all three patient cell lines.

## MSC co-culture improves mitochondrial respiration in CI-deficient fibroblasts

**Fig. 3:**
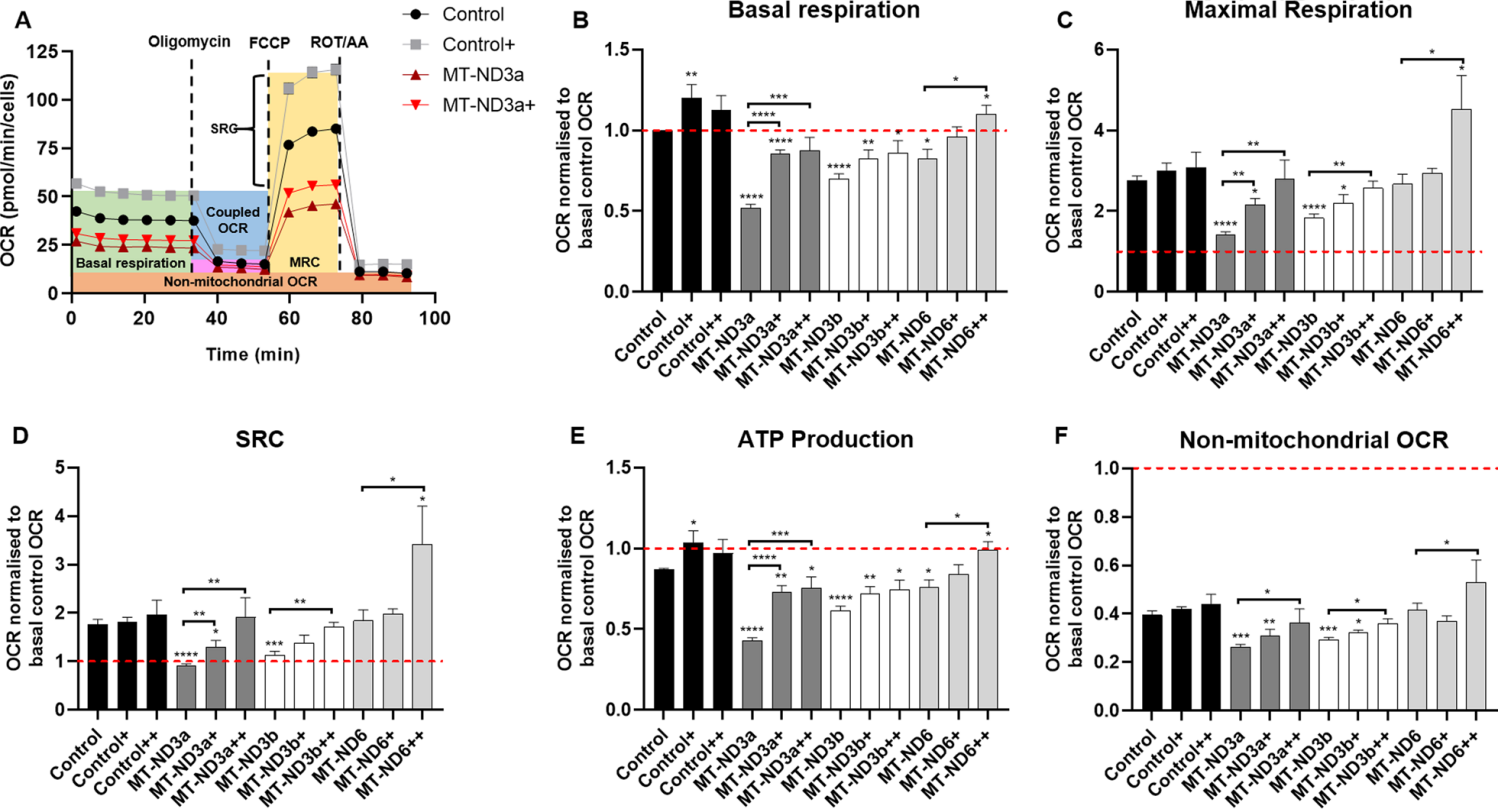
Analysis of mitochondrial respiration of healthy control and patient fibroblasts with a Seahorse XFe96 Extracellular Flux Analyzer. **A** Representative graph of a mitochondrial oxygen consumption bioenergetics profile of control (black = untreated and grey = MSC-treated) and MT-ND3a (dark red = untreated and red = MSC-treated) patient fibroblasts analysed with Mito Stress Test Assay of one experiment. First six measurements show basal respiration, followed by oligomycin injection that inhibits complex V and induces a leak state. Injections of the uncoupler FCCP stimulates the respiratory chain to increase activity to maximum by disrupting the proton gradient and mitochondrial membrane potential. Rotenone/antimycin A (Rot/AA) injection blocks respiration and enables calculation of the residual oxygen consumption. Treatment with MSCs increases overall oxygen consumption rates (OCRs) of all investigated cell lines. **B**, **C** Basal and maximal respiration of untreated MT-ND3- and MT-ND6-deficient fibroblasts compared to control fibroblasts and once (+) or twice (++) treated with MSCs. MT-ND3s show highest reduction in basal and maximal respiratory capacities (BRC and MRC, respectively) compared to control. However, all patients show decreased BRCs and MRCs. Co-cultured patient and control fibroblasts are able to increase their BRC and MRC. Sequential treatment with MSCs enhances the improvement. **D**, **E** Spare respiratory capacity (SRC) of MSC-treated (+ or ++) and untreated MT-ND3- and MT-ND6-deficient fibroblasts compared to control fibroblasts. All patients have decreased SRCs compared to control fibroblasts. Treatment with MSCs increases SRCs of all investigated cell lines, but most effectively in patient fibroblasts. Respiration-dependent ATP production is increased in all treated patient fibroblasts, with highest improvement in patient cell line MT-ND3a. **F** Non-mitochondrial oxygen consumption rates of patient fibroblasts as well as control compared to single (+) or sequential (++) treatment with MSCs show overall improvement for all three patient cell lines. OCR data were obtained in pmol/min and normalised to cell number. In a second step, data were normalised to basal respiration of untreated control (level indicated by dashed red line). Data are presented as mean ± SEM, analysed in *n* = 3 independent experiments for single and sequential treatment. All samples were checked for significance compared to untreated control and within one group (indicated by respective brackets). Statistical analysis by unpaired *t* test, *p < 0.05; **p < 0.01; ***p < 0.001; ****p < 0.0001

Mitochondrial respiration was determined by measuring the OCR with the Seahorse XFe96 Extracellular Flux Analyzer in MSC-treated fibroblasts (Fig. 3A). All three CI-deficient fibroblast lines showed a significantly reduced basal respiration compared to healthy controls (Fig. 3B). Treatment with MSCs improved basal respiration of all investigated cell lines. Sequential treatment of the fibroblasts with MSCs further enhanced the beneficial effect.

The cell’s maximum operation capacity is expressed by the maximal respiration capacity (MRC), which was determined by subtracting the maximum rate measurement after FCCP injection with the minimum rate measurement after Rot/AA injection (Fig. 3A). Again, all three CI-deficient cell lines benefited from MSC treatment regarding this parameter (Fig. 3C).

Another important parameter is the spare respiratory capacity (SRC) that is defined as the difference between maximal and basal respiration and describes the cell’s capability to react to an energetic demand (Fig. 3A). SRC was increased for all investigated patient fibroblasts (Fig. 3D). The same trend was seen for the ATP production-coupled respiration (Fig. 3E). Mitochondrial transfer to fibroblasts has also an impact on overall respiration that is not only attributed to mitochondria (Fig. 3F).

## Discussion

Transfer of mitochondria from MSCs to other cell types is a well-recognised phenomenon [11, 19]. We previously investigated this process in fibroblast lines carrying pathogenic variants in the nuclear-encoded CI subunit NDUFS4 [11]. In this experimental setting, we observed transfer of mitochondria from MSCs to NDUFS4-deficient cells, which was accompanied by a transient mitigation of ROS production. However, we observed no effect on CI abundance, which raises doubts about the functional benefits of this approach beyond antioxidative effects.

In the present study, we applied MSC co-culture to patient-derived fibroblasts with pathogenic variants in mtDNA-encoded CI subunits. We hypothesised that this setting might be more promising because MSC-based transfer of mitochondria might lead to a “genetic rescue” (e.g. transfer of the wild-type gene) that is not achieved in nuclear-encoded CI defects. Accordingly, the degree of mutation load could be lowered in patient cells, thereby mitigating the biochemical dysfunction and providing more prolonged corrective effects.

Of note, mitochondria are dynamic organelles that undergo fission and fusion events thereby exchanging intraorganellar content, which is in favour of defective mitochondria that benefit from complementation [25]. As the theory goes, mitochondrial diseases due to mtDNA mutations could be treated through exchange of genetic material between mitochondria with healthy and defective mtDNA [26, 27].

Investigation of mitochondrial transfer rate revealed similar results as previously reported by our group for the nuclear-encoded NDUFS4 defect, indicating that the genetic background of CI deficiency had no obvious impact on this phenomenon in our experimental setting. Sequencing of mtDNA was performed to further substantiate a transfer of wild-type mtDNA between fibroblasts and MSCs. To this end, we sequenced the hypervariable regions I and II of the mtDNA in MSCs as well as control and CI-deficient fibroblast. Within the hypervariable region II, we could detect up to 11 bases that were different compared to the MSC sequence. Investigation of the same positions of co-cultured fibroblasts revealed the co-existence of both, the native and the MSC mtDNA bases. This finding proves the transfer of MSC mtDNA to CI-deficient fibroblast lines.

Next, we compared the effects of MSC co-culture versus treatment with MSC supernatant on redox homeostasis in CI-deficient fibroblast lines. MSC co-culture led to a significant reduction of ROS production. Importantly, this effect was permanently present even after removal of MSCs from the culture system. A similar effect on ROS production was achieved by treatment of CI-deficient fibroblasts in a transwell co-culture system (e.g. without the possibility of direct cell–cell contact) as well as with MSC-derived supernatant. While ROS production in fibroblasts rapidly increased to pre-treatment levels after removal of MSC supernatant, ROS levels were still significantly reduced in at least two patients after transwell co-culturing. The transient mitigation of ROS production by MSC-derived medium might be linked to the high concentrations of antioxidants in MSC supernatant, which are rapidly degraded over time [28]. The results of the transwell experiments, however, indicate that additional factors/substances released by MSCs have a longer-lasting effect on redox homeostasis. These factors might have a shorter survival time in collected culture medium, which could explain the different results between the experimental settings. The above results suggest that modulation of redox homeostasis via MSC co-culture is exerted by different mechanisms than treatment with MSC supernatant alone. Direct cell–cell interactions appear to yield the most optimal result.

To further elucidate the mechanism of MSC-based ROS regulation, cellular glutathione (GSH) levels were measured. Of note, GSH is a key player in ROS detoxification. It is described that an increase in oxidative stress levels leads to accumulation of intracellular GSSG levels, which results in decreased GSH:GSSG ratio. However, we could not detect any alterations of GSH/GSSG ratio in our cell model system, which suggests that ROS is regulated on the basis of another mechanism than glutathione (see Additional files 1, 2: Fig. S1A, B).

Apart from the GSH-system, there are several other cellular antioxidant defence mechanisms. Investigation of key-antioxidant proteins revealed increased levels, which is a mitochondrial enzyme that converts oxygen radicals to H_2_O_2_. SOD2 upregulation upon oxidative stress conditions is a known phenomenon [29]. Increase in SOD2 levels is in line with elevated ROS levels in untreated patient fibroblast cell lines. Upon MSC treatment, SOD2 levels decrease, which is in keeping with the reduction of ROS levels caused by MSC treatment. Apart from SOD2, HO-1 is a protein, which is also implicated in antioxidative pathways [29]. Comparable to SOD2 (although to a lesser degree), HO-1 protein levels appeared to be moderately increased in patient-derived cell lines during untreated conditions. MSC treatment caused a reduction of HO-1 protein levels.

Besides SOD2 and HO-1, we also investigated the levels of NQO1 (NAD(P)H quinone dehydrogenase 1), a protein implicated in detoxification of redox-cycling quinones and Nrf2 a key regulator of antioxidant response element-dependent genes [29]. However, for these proteins we did not observe clear differences between patient and control cells and no obvious changes in protein levels were seen upon MSC treatment.

Our data suggest adaptive mechanisms in complex I-deficient cell lines to counteract oxidative stress. In this context, SOD2 seems to play a prominent role, which is in line with the mitochondrial origin of ROS in our model system. Interestingly, this adaptation was not clearly dependent on Nrf2, which suggests additional regulators as it was previously suggested in the literature [29]. Mitigation of oxidative stress by MSC treatment reverses the adaptation process and normalises antioxidant protein levels.

As a last step, we investigated whether an improvement of OXPHOS function is responsible for the sustained effect on ROS production and we analysed mitochondrial respiration. Experiments revealed that all CI-deficient cell lines showed a decreased basal and maximal respiratory capacity. After co-culture with MSCs, the bioenergetic profile of all cell lines significantly improved. Sequential treatment with MSCs further enhanced this effect, and respiration was elevated to control levels.

The above results demonstrate that MSC co-culture leads to a durable improvement of mitochondrial function in cell lines with mtDNA mutations. Of note, there are numerous studies in mouse models that already investigated the in vivo effects of MSC-based treatment approaches for disease conditions such as acute lung injury [14], non-alcoholic fatty liver disease [30] and peripheral neuropathy [31]. Moreover, application of MSC products to treat graft-versus-host disease is already part of clinical patient care [32]. These advances in MSC research hold promise towards translating the availability of MSC-based treatment approaches for mtDNA-encoded mitochondrial disease in the human clinical setting. In this regard, it is of particular importance that the human bone marrow-derived MSCs preparations utilised in our study were generated under current good manufacturing practice (cGMP)-compliant condition as advanced therapy medicinal products (ATMPs), thus facilitating the swift transfer of this novel therapeutic approach from bench to bedside.

## Supplementary Information

**Additional file 1.** Supplemental material and methods.

**Additional file 2. Fig. S1**: Investigation of cellular antioxidant defence systems in control and patient-derived fibroblasts after co-culturing with MSCs. A) Total GSH (GSH + GSSG) levels measured colorimetrically at 405 nm for untreated and MSC-treated (+) fibroblasts. Levels are only increased for patient MT-ND3a, which are reduced upon MSC-treatment. B) GSH:GSSG ratios under steady-state levels and after 72 h co-culture with MSCs normalised to untreated control. No differences were detected. Date are shown as mean of four independent experiments ± SEM. ** p<0.01, *** p<0.001. C–E) Representative Western blot results from nuclear and cytosolic fraction analysed for Nrf2 and loading controls and marker SDHA (mitochondrial), GAPDH (cytosolic), HDAC1 (nuclear) and α-Tubulin (cytosolic) and respective quantitative analysis from original blots of three independent experiments. Data are expressed as mean ± SD. * p<0.05.

## Data Availability

All data generated or analysed during this study are included in this published article (and its supplementary information files).

## Abbreviations

CI: Complex I
OXPHOS: Oxidative phosphorylation
mtDNA: Mitochondrial DNA
MSC: Mesenchymal stem cell
ROS: Reactive oxygen species
SOD2: Superoxide dismutase 2 mitochondrial
Nrf2: Nuclear factor erythroid 2-related factor 2
NQO1: NAD(P)H quinone dehydrogenase 1
HO-1: Heme oxygenase 1
