# Supplementary material for: Mesenchymal stem cells improve redox homeostasis and mitochondrial respiration in fibroblast cell lines with pathogenic MT-ND3 and MT-ND6 variants
Source: Stem Cell Res Ther. 2022 Jun 17;13:256. doi: 10.1186/s13287-022-02932-x (PMC9205113; doi:10.1186/s13287-022-02932-x)
Supplement: Supplementary file 1 — Additional file 1. Supplemental material and methods. [file 13287_2022_2932_MOESM1_ESM.docx]

**Supplement**

**Navaratnarajah et al. “Mesenchymal stem cells improve redox homeostasis and mitochondrial respiration in fibroblast cell lines with pathogenic *MT-ND3* and *MT-ND6* variants”**

**Supplemental material and methods**

**Glutathione colorimetric detection assay**

Total glutathione (GSH) levels and its oxidised GSH dimer (GSSG) were measured using a commercially available colorimetric detection kit (Thermo Scientific). Cells were harvested and lysed with 5 % 5-sulfo-salicylic acid dihydrate (pH 6.0) (SSA) and by five freeze-thaw cycles. Lysates were incubated 10 min on ice, followed by centrifugation (13.000 rpm, 30 min, 4°C). Half of the supernatant was treated 1 h at room temperature with 2-vinylpyridine (2-VP) to block free GSH and other thiols to determine oxidised GSH levels. All cell lysates were then diluted 1:5 with assay buffer and again 1:4 with sample diluent (assay buffer with 1 % SSA) (final dilution 1:20). Two standard series were prepared according to manufacturer’s instructions, either treated or not treated with 2-VP. Samples were applied to enclosed 96-well half area plates together with detection reagent and reaction mixture containing NADH concentrate und glutathione reductase concentrate and mixed well. After a 20 min incubation step, absorbance was measured at 405 nm. Total GSH concentration was read from the untreated standard curve, while GSSG concentration is half of the concentration read from the 2-VP-treated standard curve. The difference of both values gives concentration of free GSH in the samples. The values were expressed in µM of glutathione and were normalised to protein content of the samples. The ratio is given as free GSH concentration/GSSG concentration.

**Supplemental Figure 1: Investigation of cellular antioxidant defence systems in control and patient-derived fibroblasts after co-culturing with MSCs**

A) Total GSH (GSH + GSSG) levels measured colorimetrically at 405 nm for untreated and MSC-treated (+) fibroblasts. Levels are only increased for patient MT-ND3a, which are reduced upon MSC-treatment.
B) GSH:GSSG ratios under steady-state levels and after 72 h co-culture with MSCs normalised to untreated control. No differences were detected. Date are shown as mean of four independent experiments ± SEM. ** p<0.01, *** p<0.001.

C-E) Representative Western blot results from nuclear and cytosolic fraction analysed for Nrf2 and loading controls and marker SDHA (mitochondrial), GAPDH (cytosolic), HDAC1 (nuclear) and α-Tubulin (cytosolic) and respective quantitative analysis from original blots of three independent experiments. Data are expressed as mean ± SD. * p<0.05.
