# Supplementary figures and images for: Mesenchymal stem cells improve redox homeostasis and mitochondrial respiration in fibroblast cell lines with pathogenic MT-ND3 and MT-ND6 variants
Source: Stem Cell Res Ther. 2022 Jun 17;13:256. doi: 10.1186/s13287-022-02932-x (PMC9205113; doi:10.1186/s13287-022-02932-x)

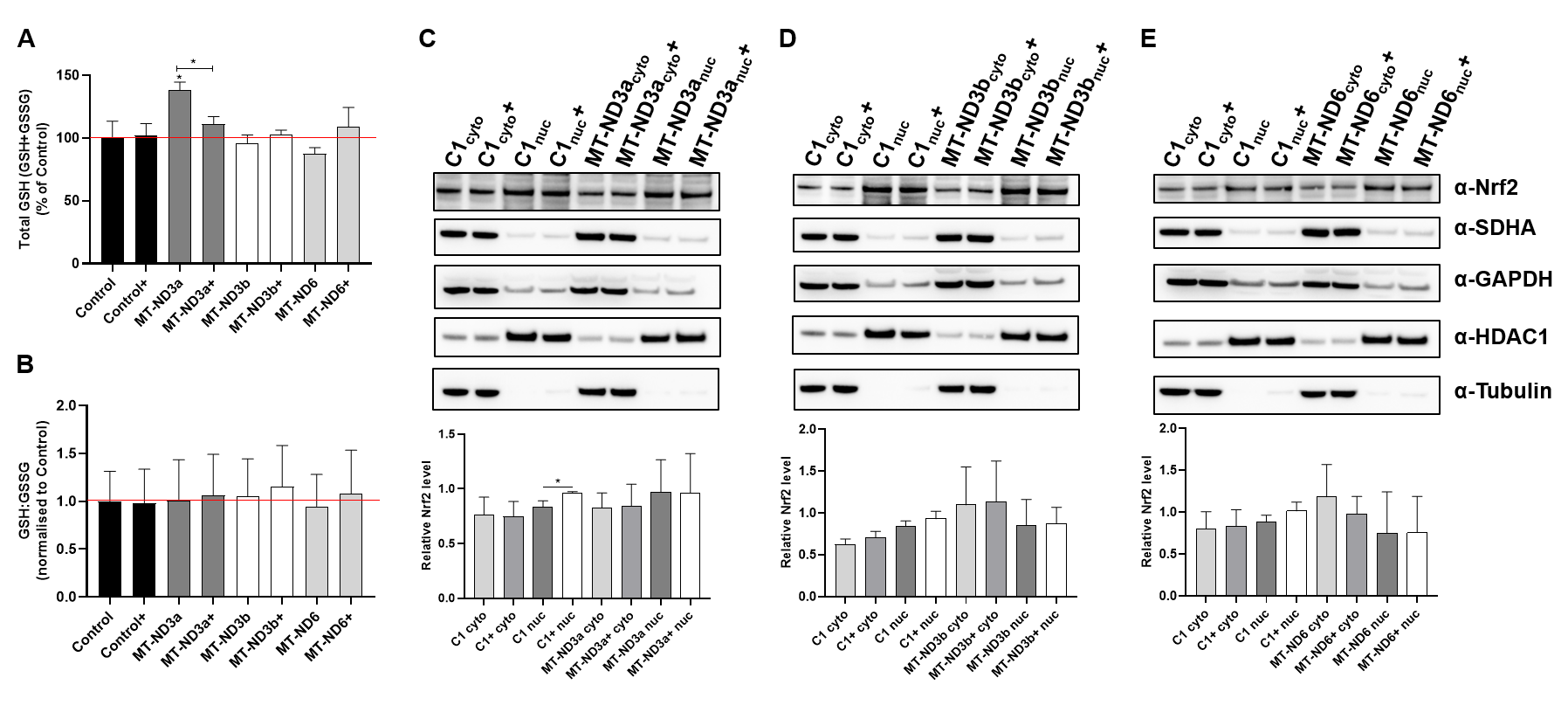

Supplement: Supplementary file 2 — Additional file 2. Fig. S1: Investigation of cellular antioxidant defence systems in control and patient-derived fibroblasts after co-culturing with MSCs. A) Total GSH (GSH + GSSG) levels measured colorimetrically at 405 nm for untreated and MSC-treated (+) fibroblasts. Levels are only increased for patient MT-ND3a, which are reduced upon MSC-treatment. B) GSH:GSSG ratios under steady-state levels and after 72 h co-culture with MSCs normalised to untreated control. No differences were detected. Date are shown as mean of four independent experiments ± SEM. ** p<0.01, *** p<0.001. C–E) Representative Western blot results from nuclear and cytosolic fraction analysed for Nrf2 and loading controls and marker SDHA (mitochondrial), GAPDH (cytosolic), HDAC1 (nuclear) and α-Tubulin (cytosolic) and respective quantitative analysis from original blots of three independent experiments. Data are expressed as mean ± SD. * p<0.05. [file 13287_2022_2932_MOESM2_ESM.tif]
